# TRPV4 Inhibition and CRISPR-Cas9 Knockout Reduce Inflammation Induced by Hyperphysiological Stretching in Human Annulus Fibrosus Cells

**DOI:** 10.3390/cells9071736

**Published:** 2020-07-21

**Authors:** Elena Cambria, Matthias J. E. Arlt, Sandra Wandel, Olga Krupkova, Wolfgang Hitzl, Fabian S. Passini, Oliver N. Hausmann, Jess G. Snedeker, Stephen J. Ferguson, Karin Wuertz-Kozak

**Affiliations:** 1 Institute for Biomechanics, ETH Zurich, 8093 Zurich, Switzerland; matthias.arlt@balgrist.ch (M.J.E.A.); sandra.wandel@datazug.ch (S.W.); olga.krupkova@usb.ch (O.K.); fabian.passini@hest.ethz.ch (F.S.P.); jess.snedeker@hest.ethz.ch (J.G.S.); sferguson@ethz.ch (S.J.F.); kwbme@rit.edu (K.W.-K.); 2 Department of Orthopedics, Balgrist University Hospital, University of Zurich, 8008 Zurich, Switzerland; 3 Department of Biomedicine, University of Basel, 4031 Basel, Switzerland; 4 Spine Surgery, University Hospital Basel, 4031 Basel, Switzerland; 5 Research Office (Biostatistics), Paracelsus Medical University, 5020 Salzburg, Austria; wolfgang.hitzl@pmu.ac.at; 6 Department of Ophthalmology and Optometry, Paracelsus Medical University, 5020 Salzburg, Austria; 7 Research Program Experimental Ophthalmology and Glaucoma Research, Paracelsus Medical University, 5020 Salzburg, Austria; 8 Neuro- and Spine Center, Hirslanden Klinik St. Anna, 6006 Lucerne, Switzerland; ohausmann@hin.ch; 9 Medical Faculty, University of Bern, 3012 Bern, Switzerland; 10 Department of Biomedical Engineering, Rochester Institute of Technology, Rochester, NY 14623, USA; 11 Spine Center, Schön Klinik München Harlaching, Academic Teaching Hospital and Spine Research Institute of the Paracelsus Private Medical University Salzburg (Austria), 81547 Munich, Germany

**Keywords:** mechanotransduction, cyclic stretching, transient receptor potential channel, gene editing, interleukins, low back pain

## Abstract

Mechanical loading and inflammation interact to cause degenerative disc disease and low back pain (LBP). However, the underlying mechanosensing and mechanotransductive pathways are poorly understood. This results in untargeted pharmacological treatments that do not take the mechanical aspect of LBP into account. We investigated the role of the mechanosensitive ion channel TRPV4 in stretch-induced inflammation in human annulus fibrosus (AF) cells. The cells were cyclically stretched to 20% hyperphysiological strain. TRPV4 was either inhibited with the selective TRPV4 antagonist GSK2193874 or knocked out (KO) via CRISPR-Cas9 gene editing. The gene expression, inflammatory mediator release and MAPK pathway activation were analyzed. Hyperphysiological cyclic stretching significantly increased the IL6, IL8, and COX2 mRNA, PGE2 release, and activated p38 MAPK. The TRPV4 pharmacological inhibition significantly attenuated these effects. TRPV4 KO further prevented the stretch-induced upregulation of IL8 mRNA and reduced IL6 and IL8 release, thus supporting the inhibition data. We provide novel evidence that TRPV4 transduces hyperphysiological mechanical signals into inflammatory responses in human AF cells, possibly via p38. Additionally, we show for the first time the successful gene editing of human AF cells via CRISPR-Cas9. The pharmacological inhibition or CRISPR-based targeting of TRPV4 may constitute a potential therapeutic strategy to tackle discogenic LBP in patients with AF injury.

## 1. Introduction

Unlocking the mechanisms leading from mechanical stimuli to cellular sensing and response has become necessary to understand and tackle diseases in essentially all medical disciplines [1]. Musculoskeletal disorders are often associated with abnormal mechanical stress applied to load-bearing tissues [2,3]. In the intervertebral disc (IVD), compressive forces from the body weight and spinal motions generate hydrostatic and osmotic pressures in the central gelatinous nucleus pulposus, which in turn increase tensile stresses in the ring-shaped annulus fibrosus (AF) [4]. While physiological mechanostimulation is favorable and even necessary to maintain IVD homeostasis, hyperphysiological mechanical loading is a well known contributor to IVD degeneration [5]. Furthermore, mechanical overloading interacts with inflammation and catabolism to cause degenerative disc disease (DDD) [6,7,8,9]. DDD, characterized by IVD structural failure and nociception [6], is the main cause of discogenic back pain. Low back pain (LBP) is a condition with a lifetime prevalence of 84% and the primary cause of disability worldwide [10,11]. In particular, AF disruption is commonly linked to LBP and disability [12]. Current pharmacological treatments of LBP, such as anti-inflammatory drugs, do not take into account the mechanical aspect of the problem. Moreover, they have low effect sizes and their mechanisms of action remain unclear [10]. A profound knowledge of the mechanotransductive pathways leading to the degeneration and inflammation of the AF could improve the efficacy of novel pharmaceutical therapies.

Several studies have previously proved the detrimental effects of hyperphysiological mechanical loading on IVD cells at the molecular level. Cyclic stretching of IVD (mostly AF) cells at a high strain from 8 to 20% was shown to induce the downregulation of anabolic markers (ACAN, COL2) [13] and the upregulation of catabolic (MMP1, MMP3, MMP9, MMP13, ADAMTS4, ADAMTS5) [13,14] and (pro-)inflammatory (COX2, PGE2, IL1β, IL6, IL8, IL15, TLR2, TLR4, NGF, TNFα, MCP1, MCP3, MIG) [13,14,15,16,17] mediators. Stretch-induced inflammation in the IVD may be regulated by the NF-κB [13] or the mitogen-activated protein kinase (MAPK: ERK1/2, p38 and JNK) [17] signaling pathways. Nevertheless, the exact mechanosensing and mechanotransductive mechanisms of AF cells remain poorly understood.

Transient receptor potential (TRP) ion channels have emerged as a novel class of cellular sensors and as potential therapeutic targets to treat several diseases [18]. TRP channels are non-selective calcium-permeable transmembrane channels that are sensitive to a variety of stimuli, including temperature, pH, oxidative stress, and importantly, mechanical stress [19]. There are six sub-families of mammalian TRP channels: TRPC (canonical), TRPV (vanilloid), TRPM (melastatin), TRPP (polycystin), TRPML (mucolipin) and TRPA (ankyrin) [19]. The member 4 of the vanilloid subfamily (TRPV4) is of particular interest in the context of joint diseases, due to its ability to transduce mechanical, inflammatory and pain signals [20]. In chondrocytes, similar in many aspects to IVD cells, TRPV4 was shown to mediate the transduction of dynamic compressive loading [21]. Moreover, cartilage-specific TRPV4 knockout in mice reduced age-related osteoarthritis [22]. The exploration of TRP channels in the IVD is at its infancy, with only few published studies [23,24,25,26,27]. Most of the known TRP channel genes are expressed in the IVD [26]. Interestingly, TRPV4 has been associated with reduced osmolarity and pro-inflammatory cytokines in bovine nucleus pulposus cells [23]. Additionally, treatment with IL1β increases TRPV4 gene expression in human IVD cells, thus revealing a potential inflammatory role of the channel in IVDs [27]. Nevertheless, the roles of TRPV4 in mechanosensing and mechanotransduction in the IVD remain to be established.

In this study, we aimed to investigate whether stretch-induced inflammation is signaled through TRPV4 in human primary AF cells. To this end, we modulated the TRPV4 activity and expression through pharmacological inhibition and CRISPR-Cas9 knockout, respectively, in AF cells that were cyclically stretched at a hyperphysiological strain of 20%.

## 2. Materials and Methods

### 2.1. Human AF Cell Isolation and Culture

Human IVD biopsies were obtained from patients undergoing spinal surgery for disc herniation or DDD after informed consent. Patient characteristics can be found in Table 1. The study was conducted in accordance with the Declaration of Helsinki, and the protocol was approved by the ethical committee of the Canton of Zurich, Switzerland (2019-00736). AF tissue was intraoperatively excised from the IVD and enzymatically digested overnight at 37 °C, 5% CO_2_, using 0.2% collagenase NB4 (17454, Serva) and 0.3% dispase II (04942078001, Roche) in PBS 1X with 5% antibiotic–antimycotic (anti–anti; 15240-062, Gibco, Switzerland). Isolated cells were cultured up to passage 1–2 at 37 °C, 5% CO_2_ in a growth medium (Dulbecco’s modified Eagle medium/F-12 Nutrient Mixture (DMEM/F12; 31330-038, Gibco, Switzerland), supplemented with 10% fetal calf serum (FCS; F7524, Sigma-Aldrich, Switzerland) and 1% anti–anti).

### 2.2. Generation of CRISPR-Cas9 TRPV4 Knockout (KO) Cells

The CRISPRdirect online tool (http://crispr.dbcls.jp; [28]) was used to design a single guide RNA (sgRNA) against TRPV4 with a highly specific target site (TRPV4 (hu) 739–761: CGGAGCGCACCGGCAACATG). A non-targeting control sgRNA (Hu Non-Targeting 40: GACTTATAAACTCGCGCGGA) was chosen from the study of Morgens et al. [29]. Low targeting potential was checked by a BLASTN (BLASTN 2.8.0) search. Target sequences’ oligos were synthesized with BsmBI restriction site overhangs by Microsynth (Balgach, Switzerland) and then annealed and cloned into the lentiCRISPRv2 transfer plasmid, a gift from Feng Zhang (Addgene plasmid # 52961; [30]), according to the protocol of the Feng Zhang Lab. 

HEK293T cells were co-transfected with the lentiCRISPRv2 plasmid with the packaging plasmids pCMV-VSV-G (a gift from Bob Weinberg; Addgene plasmid # 8454; [31]) and psPAX2 (a gift from Didier Trono; Addgene plasmid # 12260) using Lipofectamine 3000 (Invitrogen, Thermo Fisher Scientific) according to the manufacturer’s instructions, in order to produce lentiviral particles.

Human AF cells were transduced through incubation with medium containing viral particles and 8 µg/mL polybrene for 24 h. The cells were selected with 4 µg/mL puromycin (A1113803, Gibco) for 7 days and cultured for 8 weeks in growth medium before being tested. The efficiency of the KOs was tested by RT-qPCR and immunocytochemistry.

### 2.3. Cyclic Stretching

Commercial stretching chambers (10 cm^2^, STB-CH-10, STREX) were coated with 50 µg/mL fibronectin (FC010, Merck Millipore) overnight at 37 °C. The human AF cells at passage 1–2 were seeded in the chambers (100,000 cells per chamber in 5 mL growth medium) and cultured for 72 h at 37 °C, 5% CO_2_, unless otherwise stated. The cells were serum-starved for 4–12 h in a no-serum medium (DMEM/F12 with 0.1% ampicillin (A0839, AppliChem, Darmstadt, Germany). The chambers were mounted on a commercial stretching bioreactor (STB-140-10, STREX) and subjected to 20% cyclic sinusoidal uniaxial strain at a frequency of 1 Hz at 37 °C and 5% CO_2_. Control chambers were kept in identical conditions without stretching.

Time course: Cells were stretched for 1, 2, 4, 8, 12 or 24 h. The stretching started with the chambers that were stretched for 24 h, and shorter duration chambers were added progressively on the device so that the stretching was stopped at the same time for all conditions. Immediately after the mechanical loading, the cells were lysed for gene expression analysis.

TRPV4 inhibition: Cells were pre-treated for 15 min with 0, 20, 50, 100, 200 or 500 nM of the selective TRPV4 antagonist GSK2193874 (1336960-13-4, Sigma-Aldrich). The concentration of the vehicle (DMSO) was equalized in all chambers (0.005%). Cells were subjected to cyclic stretching for 1 h in the presence of the antagonist or vehicle and either immediately lysed for gene expression analysis or cultured in 2 mL of fresh no-serum medium for 24 h for analysis of the conditioned medium. 

MAPK activation: Cells were seeded in the chambers (300,000 cells per chamber in 5 mL growth medium), cultured for 24 h at 37 °C, 5% CO_2_ and serum-starved. Cells were pre-treated for 15 min with 0, 20, 100, 200 or 500 nM of GSK2193874 and stretched for 15 min in the presence of the antagonist or vehicle. As a positive control, the cells were pre-treated for 15 min with 10 ng/mL recombinant human IL1β (200-01B, PeproTech) and further incubated for 15 min without stretching in the presence of IL1β. Cells were lysed for Western blot immediately after stretching.

TRPV4 knockout: CRISPR-Cas9-transduced non-targeting control (NT) and TRPV4 knockout (KO) cells were subjected to cyclic stretching for 1 h and either immediately lysed for gene expression analysis or cultured in 2 mL of fresh no-serum medium for 24 h for analysis of the conditioned medium.

### 2.4. RNA Extraction and RT-qPCR

Immediately after stretching, the cells were rinsed with cold PBS 1X and lysed on ice with 600 µL per chamber of GENEzol (GZR200, Geneaid) using a cell scraper. The RNA was extracted with chloroform, precipitated with isopropanol, washed in ethanol and resuspended in RNase-free water according to the Geneaid instructions. The RNA yield and purity were measured on a NanoDrop 1000 Spectrophotometer (Thermo Fisher Scientific). The TaqMan Reverse Transcription kit (N8080234, Applied Biosystems) was used to reverse-transcribe 1 μg of RNA into cDNA in a 30 µL volume. PCR reactions were carried out in a 10 µL total volume containing 20 ng of cDNA, TaqMan primers (TRPV4: Hs01099348_m1; COL1A1: Hs00164004_m1; COL2A1: Hs00264051_m1; ACAN: Hs00153936_m1; MMP1: Hs00233958_m1; MMP3: Hs00968308_m1; MMP13: Hs00233992_m1; IL6: Hs00174131_m1; CXCL8 (IL8): Hs00174103_m1; PTGS2 (COX2): Hs00153133_m1; SDHA: Hs00188166_m1), TaqMan Fast Universal PCR Master Mix (2X) (4352042, Applied Biosystems) and RNase-free water. To measure the TRPV4 mRNA expression of the CRISPR-Cas9 transduced cells, primers (hu TRPV4-652-F: CATCTACGGGGAAGACCTGC; hu TRPV4-768-R: TGAACTCCCTCATGTTGCCG; hu ANXA5-633-F: CCTTCAGGCTAACAGAGACCC; hu ANXA5-728-R: CCCCATTTAAGTTCTCCAGCC) were mixed with 10 ng cDNA, Power SYBR Green Master Mix (2X) (4368577, Applied Biosystems) and RNase-free water in a total volume of 20 µL. The CFX96 Touch Detection System (Bio-Rad) was used to measure the gene expression in duplicates. SDHA (or ANXA5) was used as a reference gene. Results are shown as 2^−ΔΔCt^ values (i.e., relative to SDHA (or ANXA5) and the control condition).

### 2.5. ELISA of Conditioned Medium

ELISA kits were used to quantify the levels of IL6, IL8 (Human IL-6 ELISA set, 555220; Human IL-8 ELISA set, 555244; Reagent set B, 550534, BD OptEIA, BD Biosciences) and PGE2 (RD-PGE2-Ge, Reddot Biotech) in the conditioned medium according to the manufacturer’s instructions. The samples used were undiluted.

### 2.6. Western Blot

The cells were rinsed with cold PBS 1X and lysed on ice with 100 µL per chamber of radioimmunoprecipitation assay (RIPA) buffer supplemented with phosphatase and protease inhibitor cocktails (89900, 78428, 78430, Thermo Fisher Scientific) using a cell scraper. The lysates were mixed with 4X Laemmli buffer (1610747, Bio-Rad), heated for 5 min at 95 °C, and loaded onto a 4–20% gradient gel (4568093, Bio-Rad). The proteins were separated by electrophoresis in a Mini-PROTEAN Tetra Cell (Bio-Rad) and transferred to a PVDF membrane (1704156, Bio-Rad). The membranes were washed for 3 × 10 min with Tris buffered saline with 0.05% TWEEN 20 (TBS-T) and blocked for 2 h at room temperature (RT) with 5% skim milk diluted in TBS-T. Primary antibodies (ERK1/2 (ERK): 9102; p-ERK: 9101; p38: 9212; p-p38: 9211; JNK: 9252; p-JNK: 9251; α-tubulin: 2144; Cell Signaling Technology) were applied 1:1000 in 3% bovine serum albumin (BSA) in TBS-T overnight at 4 °C on a rocker. The next day, the membranes were washed for 3 × 10 min with TBS-T and incubated for 1 h at RT on a rocker with the secondary antibody (anti-rabbit IgG HRP: 7074, Cell Signaling Technology) diluted 1:3000 in 5% skim milk in TBS-T. After washing for 3 × 10 min with TBS-T, the proteins were detected with a chemiluminescence substrate (34580, Thermo Fisher Scientific) on a ChemiDocTouch Imaging System (Bio-Rad). The density of the bands was semi-quantified using the ImageLab 6.0 software (Bio-Rad). For each blot, the density of each band was normalized by the one of the non-stretched control (or the stretched 0 nM condition for JNK and p-JNK, since there were no p-JNK bands for the non-stretched control). Phosphorylated targets were expressed as relative to total targets.

### 2.7. Immunocytochemistry

Two-well chambered coverglasses (155380, Thermo Fisher Scientific) were coated with 10 µg/mL fibronectin for 30 min at 37 °C. The CRISPR-Cas9 transduced cells (NT and KO) were seeded (60,000 cells/well) in growth medium and incubated for 24 h at 37 °C, 5% CO_2_. The cells were washed with PBS 1X and fixed for 10 min with methanol at −20 °C. After washing with PBS, the cells were blocked with 5% normal goat serum (G9023, Sigma) in PBS for 1 h at RT. The primary antibody (rabbit polyclonal anti-TRPV4, ACC-034, Alomone Labs) was diluted 1:500 in 1% normal goat serum in PBS and applied overnight at 4 °C with rocking. For the no primary antibody control (negative control), 1% normal goat serum was applied on NT cells. The cells were washed extensively with PBS and incubated for 1 h at RT with the secondary antibody (Cy2 goat anti-rabbit IgG, 111-225-144, Jackson ImmunoResearch) diluted 1:200 in 1% normal goat serum in PBS. After washing with PBS, the samples were mounted with antifade mountant with 4′,6-diamidino-2-phenylindole (DAPI) (P36962, Thermo Fisher Scientific) and cured for 24 h at RT. The samples were imaged with an Olympus IX51 microscope and a 20× objective.

### 2.8. Statistical Analysis

The data were checked for consistency and for normality using skewness, kurtosis and omnibus tests. Due to the small sample sizes, the dependent bootstrap t-test based on 100,000 Monte Carlo samples were used. In addition, the classical dependent t-tests and nonparametric tests (Wilcoxon-Signed test) and the Quantile Sign test were used. All the reported tests were two-sided, and the *p*-values below 0.05 were considered statistically significant. All the statistical analyses were performed with NCSS (NCSS 10, NCSS, LLC. Kaysville, UT) and the PASW 24 (IBM SPSS Statistics for Windows, Version 21.0., Armonk, NY).

## 3. Results

### 3.1. Hyperphysiological Cyclic Stretching Induces Gene Expression of Pro-Inflammatory Mediators in Human AF Cells

**Figure 1:**
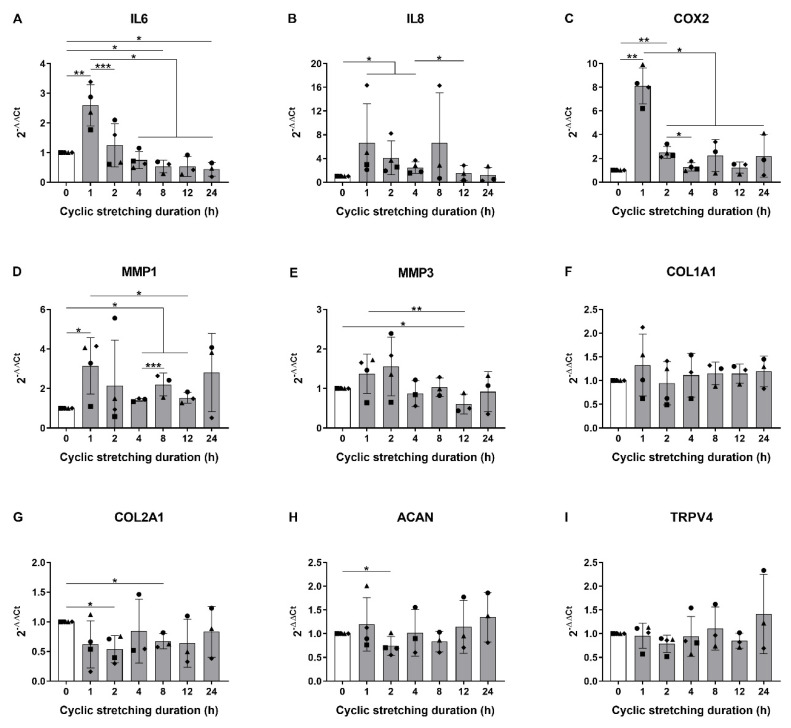
Gene expression of (**A**–**C**) pro-inflammatory mediators; (**D**,**E**) matrix metalloproteinases; (**F**–**H**) extracellular matrix; and (**I**) TRPV4 immediately after 0 (white bar) or from 1 to 24 h (grey bars) of cyclic stretching at 20% strain and 1 Hz. *n* = 3–4 donors; mean ± SD; * *p* < 0.05, ** *p* < 0.01, *** *p* < 0.001.

In a first step, we analyzed the influence of increasing the duration of cyclic stretching on the gene expression of key mediators such as pro-inflammatory, catabolic and anabolic markers, as well as TRPV4 in human AF cells. A stretching frequency of 1 Hz was chosen to imitate the walking frequency, and a 20% strain was selected as a hyperphysiological magnitude [4]. The shortest duration of 1 h considerably increased the mRNA levels of interleukin 6 (IL6; 2.60-fold), interleukin 8 (IL8; 6.62-fold), cyclooxygenase 2 (COX2; 8.11-fold) and matrix metalloproteinase 1 (MMP1; 3.15-fold) compared to the non-stretched controls (Figure 1A–D). The gene expression of matrix metalloproteinase 3 (MMP3) slightly augmented (1.37-fold) after 1 h stretching, but this change was not significant. Matrix metalloproteinase 13 (MMP13) was detected in only one donor out of three (not shown), and this gene was thus excluded from the statistical analysis. Interestingly, the increase in pro-inflammatory and catabolic mediators at 1 h was then generally dampened with increasing stretching durations (Figure 1A–E), suggesting a fast and acute response to mechanical loading. Regarding extracellular matrix genes, while the expression of collagen I (COL1A1) remained unchanged (Figure 1F), the collagen II (COL2A1) and aggrecan (ACAN) levels were decreased (0.54- and 0.74-fold, respectively) at a later time point of 2 h stretching compared to the control (Figure 1G,H). No significant changes were observed in the TRPV4 gene expression (Figure 1I). With the increased gene expression of IL6, IL8, COX2 and MMP1 at 1 h and the reduced expression of COL2A1 and ACAN at 2 h, we established a model of acute pro-inflammatory response to hyperphysiological stretching typical for early-stage AF injury [12]. 

### 3.2. Pharmacological Inhibition of TRPV4 Reduces Stretch-Induced Gene Expression of Pro-Inflammatory Mediators

**Figure 2:**
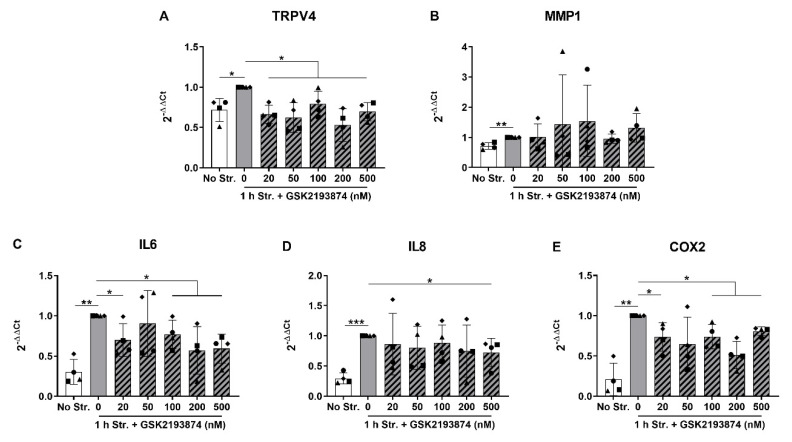
Gene expression of (**A**) TRPV4; (**B**), MMP1; and (**C**–**E**) pro-inflammatory mediators immediately after no (white bar) or 1 h (grey bars) of cyclic stretching at 20% strain and 1 Hz in the absence or presence (hatched bars) of 20–500 nM of the TRPV4 antagonist GSK2193874. *n* = 4 donors; mean ± SD; * *p* < 0.05, ** *p* < 0.01, *** *p* < 0.001.

In order to investigate the potential role of the TRPV4 ion channel in the increased expression of IL6, IL8, COX2 and MMP1 induced by hyperphysiological stretching, we selected the stretching duration of 1 h, and further cyclically stretched AF cells in the absence or presence of the selective TRPV4 antagonist GSK2193874 (20 to 500 nM). The non-stretched experimental condition was kept as a benchmark, and the concentration of the vehicle (DMSO) was equalized in all conditions (0.005%). The control cells stretched without antagonist showed a slight augmentation in the TRPV4 mRNA compared to the non-stretched cells in this data set (Figure 2A). All the concentrations of GSK2193874 moderately reduced the gene expression of TRPV4 compared to the 0 nM control condition (Figure 2A). MMP1 gene expression was only slightly but significantly increased by 1 h stretching compared to the non-stretched cells (Figure 2B), but the TRPV4 modulation did not affect this change (Figure 2B). The expression of IL6, IL8 and COX2 was confirmed to be significantly increased by 1 h cyclic stretching compared to the non-stretched cells (Figure 2C–E). Remarkably, these stretch-induced changes were significantly mitigated by the TRPV4 pharmacological inhibition (at 20 and 100 to 500 nM of GSK2193874 for IL6 and COX2, and 500 nM for IL8; Figure 2C–E). These data suggest that TRPV4 partially mediates the stretch-induced gene expression of IL6, IL8 and COX2, but not MMP1.

### 3.3. Pharmacological Inhibition of TRPV4 Downregulates the Release of IL8 and PGE2

**Figure 3:**
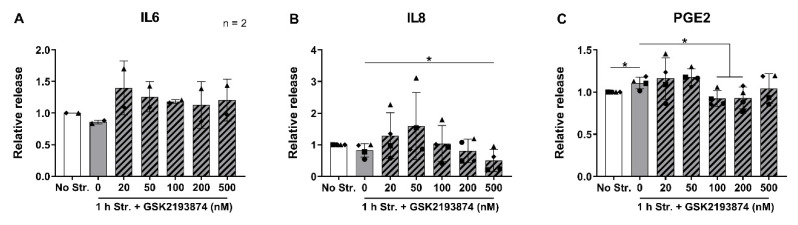
Relative release of (**A**) IL6; (**B**) IL8; and (**C**) PGE2 24 h after no (white bar) or 1 h (grey bars) of cyclic stretching at 20% strain and 1 Hz in the absence or presence (hatched bars) of 20–500 nM of the TRPV4 antagonist GSK2193874. *n* = 4 donors (*n* = 2 for IL6); mean ± SD; * *p* < 0.05, ** *p* < 0.01, *** *p* < 0.001.

In a next step, the cells stretched for 1 h with or without GSK2193874, were further cultured for 24 h, in order to measure the release of the pro-inflammatory mediators IL6, IL8 and prostaglandin E2 (PGE2, a product of COX2). The concentrations of these mediators in the conditioned medium of non-stretched samples varied between donors: with a mean of 8.46 ± 11.90 (SD) pg/mL for IL6, 13.50 ± 9.67 pg/mL for IL8, and 9.49 ± 2.22 pg/mL for PGE2. Two donors out of four released concentrations of IL6 below the limit of detection of the assay. Surprisingly, no changes in the IL6 or IL8 release due to stretching were observed (Figure 3A,B). Nevertheless, the samples treated with 500 nM GSK2193874 during stretching exhibited a lower release of IL8 compared to the samples stretched in the absence of the antagonist (Figure 3B). The release of PGE2 slightly but significantly increased in the stretched samples compared to the controls, and was further attenuated by 100 and 200 nM of the TRPV4 inhibitor (Figure 3C). These data thus show that TRPV4 inhibition decreases IL8 release and stretch-induced PGE2 release.

### 3.4. Pharmacological Inhibition of TRPV4 Reduces Stretch-Induced p38 Phosphorylation

**Figure 4:**
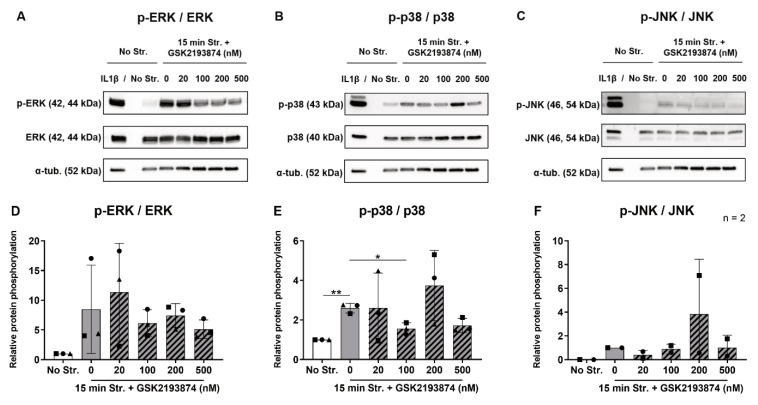
Representative Western blots from one donor (**A**–**C**) and densitometry analysis (**D**–**F**) of the phosphorylated and total (**A**,**D**) ERK 1/2; (**B**,**E**) p38; and (**C**,**F**) JNK immediately after no (white bar) or 15 min (grey bars) of cyclic stretching at 20% strain and 1 Hz in the absence or presence (hatched bars) of 20–500 nM of the TRPV4 antagonist GSK2193874. Non-stretched cells treated with IL1β were used as a positive control for the blots. One same blot of α-tubulin is shown three times as a loading control. *n* = 3 donors (*n* = 2 for p-JNK); mean ± SD; * *p* < 0.05, ** *p* < 0.01.

Cyclic stretching was previously shown to stimulate the gene expression of IL6, IL8 and COX2 via the phosphorylation of the extracellular signal-regulated kinases 1/2 (ERK), p38 and Jun-N-terminal kinase (JNK) in human AF cells [17]. In order to explore whether TRPV4 mediates the stretch-induced activation of MAPKs, we measured the expression of total and phosphorylated MAPKs after 15 min of stretching in the absence or presence of the TRPV4 antagonist. Non-stretched cells were added as a negative control, and the non-stretched cells treated with 10 ng/mL IL1β served as a positive control of the inflammatory response. Treatment with IL1β triggered a strong phosphorylation of ERK, p38 and JNK in all donors (Figure 4A–C). On the contrary, the bands for p-ERK and p-p38 were very faint (Figure 4A,B), and even absent for p-JNK (Figure 4C) in the non-stretched control samples. The expression of the total targets ERK, p38 and JNK remained similar across the conditions (Figure 4A–C). The samples cyclically stretched for 15 min without the TRPV4 antagonist displayed denser bands for all the phosphorylated MAPKs compared to non-stretched controls in all donors (Figure 4A–C). Upon the analysis of the densitometry data, even though ERK showed an 8.49-fold increase in activation in stretched samples compared to the controls (Figure 4D), only the 2.60-fold upregulation of the p38 phosphorylation was statistically significant (Figure 4E). One donor out of three expressed phosphorylated JNK in the positive control, but not in the other experimental conditions, thus preventing statistical analysis (Figure 4F). Remarkably, stretch-induced p38 phosphorylation was reduced by 100 nM GSK2193874 (Figure 4E). Although the treatment with the TRPV4 antagonist often yielded dimmer bands compared to the 0 nM condition for p-ERK and p-JNK (Figure 4A,C), these differences were not significant (Figure 4D,F). These experiments show that hyperphysiological cyclic stretching induces the phosphorylation of p38, and this is partially mediated by TRPV4.

### 3.5. CRISPR-Cas9 Knocks Out TRPV4 in Human Primary AF Cells

**Figure 5:**
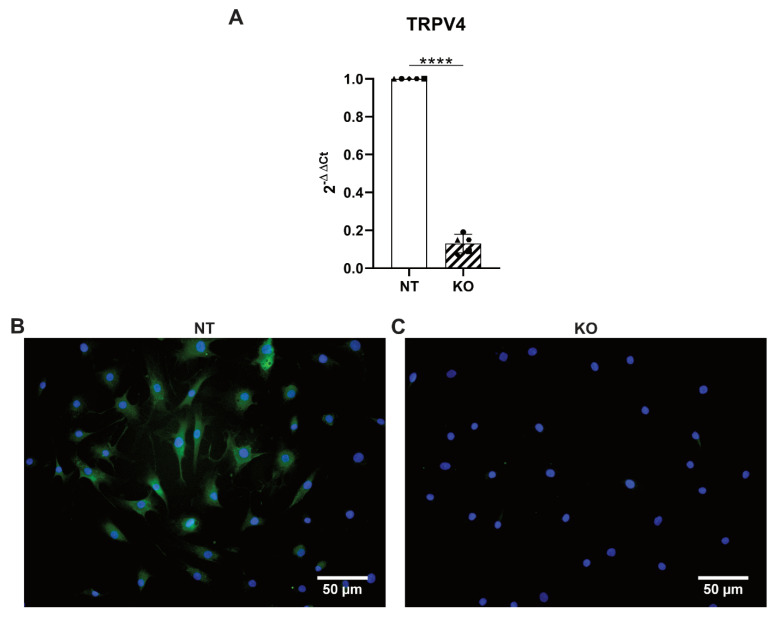
(**A**) Gene expression of TRPV4 in the CRISPR-Cas9-transduced non-targeting control (NT, white bar) and the TRPV4 knockout (KO, hatched bar) cells. *n* = 5 donors; mean ± SD; **** *p* < 0.0001. Immunocytochemistry of (**B**) the NT and (**C**) the KO cells; green = TRPV4, blue = DAPI; scale bars = 50 µm.

In a second part of the study, CRISPR-Cas9 TRPV4 KO cells were generated to further investigate the role of TRPV4 in the stretching-mediated cellular response. Human AF cells were transduced with lentiviral particles containing either a non-targeting sgRNA (NT cells) or a sgRNA against TRPV4 (KO cells). The efficiency of the KOs was tested with RT-qPCR and immunocytochemistry. The gene expression of TRPV4 significantly dropped in the KO cells to 0.13-fold compared to the NT controls, thus revealing a KO efficiency of 87% (Figure 5A). While the NT cells stained without primary antibody showed virtually no signal of TRPV4 (Figure S1), the NT cells that followed the full immunostaining protocol mostly displayed a robust expression of the ion channel (Figure 5B). At the same time, the KO cells expressed TRPV4 at a very low level similar to the no primary antibody control (Figure 5C). We thus show for the first time to our knowledge the gene editing of human AF cells via CRISPR-Cas9. The KO of TRPV4 was successful and confirmed both at the gene and protein levels.

### 3.6. CRISPR-Cas9 KO of TRPV4 Prevents Stretch-Induced Gene Expression of IL8

**Figure 6:**
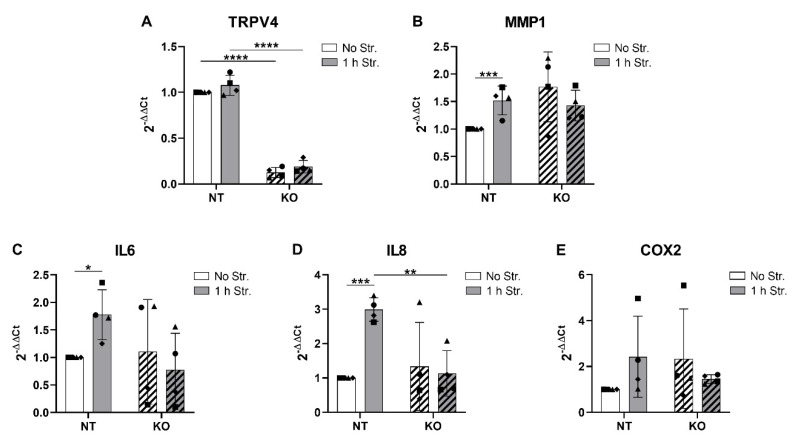
Gene expression of (**A**) TRPV4; (**B**) MMP1; and (**C**–**E**) pro-inflammatory mediators in the CRISPR-Cas9-transduced non-targeting control (NT, non-hatched bars) and the TRPV4 knockout (KO, hatched bars) cells, immediately after no (white bars) or 1 h (grey bars) of cyclic stretching at 20% strain and 1 Hz. *n* = 4 donors; mean ± SD; * *p* < 0.05, ** *p* < 0.01, *** *p* < 0.001, **** *p* < 0.0001.

Similar to the TRPV4 inhibition experiments, the CRISPR-Cas9-transduced NT and TRPV4 KO cells were subjected to hyperphysiological cyclic stretching for 1 h and immediately lysed for gene expression analysis. The TRPV4 mRNA levels in the NT and KO cells were not affected by mechanical loading (Figure 6A). While the MMP1 gene expression was once again significantly augmented (1.52-fold) by stretching compared to the non-stretched controls in the NT cells, this change was not observed in the TRPV4 KO cells (Figure 6B). However, TRPV4 KO non-stretched cells showed variable and relatively high levels of MMP1 (Figure 6B). As expected, IL6 and IL8 gene expression was significantly upregulated (1.78- and 2.99-fold, respectively) by stretching in NT cells (Figure 6C,D). Although IL6 mRNA levels varied among donors in TRPV4 KO non-stretched cells, these were either reduced or remained constant upon stretching (Figure 6C). The decrease in IL6 in TRPV4 KO stretched cells compared to NT stretched cells was nonetheless not statistically significant (Figure 6C). The stretch-induced upregulation of the IL8 gene expression was completely prevented in TRPV4 KO cells (Figure 6D). Surprisingly, the gene expression of COX2 was not significantly altered by stretching in NT cells, and no changes were observed in TRPV4 KO cells (Figure 6E). These data support the findings obtained with the TRPV4 inhibition, in that TRPV4 KO prevented the stretch-induced upregulation of IL8 mRNA and tended to reduce the stretch-induced IL6 mRNA.

### 3.7. CRISPR-Cas9 KO of TRPV4 Downregulates the Release of IL6 and IL8

**Figure 7:**
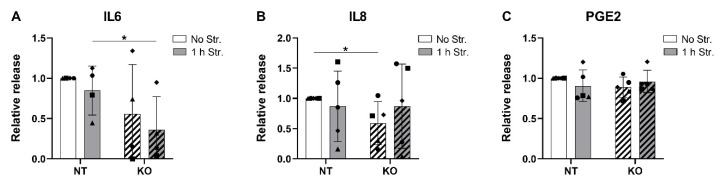
Relative release of (**A**) IL6; (**B**) IL8; and (**C**) PGE2 in the CRISPR-Cas9-transduced non-targeting control (NT, non-hatched bars) and the TRPV4 knockout (KO, hatched bars) cells 24 h after no (white bars) or 1 h (grey bars) of cyclic stretching at 20% strain and 1 Hz. *n* = 4–5 donors; mean ± SD; * *p* < 0.05.

In a final step, we analyzed the release of IL6, IL8 and PGE2 in the conditioned medium of the NT and TRPV4 KO cells that were cyclically stretched for 1 h and cultured for 24 h. The basal concentrations of IL6, IL8 and PGE2 in the NT non-stretched cells varied between donors: 146.3 ± 145.7 pg/mL, 170.9 ± 160.8 pg/mL, and 12.80 ± 8.01 pg/mL, respectively. One donor out of five released concentrations of IL6 below the limit of detection of the assay. Similar to the TRPV4 inhibition experiment, the stretch-induced upregulation of the IL6 and IL8 genes in NT cells did not result in an increase in IL6 and IL8 release in the conditioned medium (Figure 7A,B). Nevertheless, the release of IL6 was significantly lower in the KO stretched cells compared to the NT stretched cells (Figure 7A). This effect was not observed for IL8, but the KO non-stretched cells released less IL8 compared to their NT counterparts (Figure 7B). No significant changes were observed in the PGE2 release across conditions (Figure 7C). These data support the link between TRPV4 and IL8 release, and suggest a role of TRPV4 in mediating IL6 release.

## 4. Discussion

Treating diseases in load-bearing tissues often requires an extensive knowledge of the interaction between the mechanical and inflammatory signals, a concept recently coined as “mechanoflammation” [32]. Understanding the mechanosensing and mechanotransductive pathways at the origin of DDD and LBP would contribute to finding targeted treatments and thus, lightening the immense burden carried by society and the health economy.

In this study, we report novel evidence that mechanoinflammatory responses induced by hyperphysiological stretching are significantly mitigated by TRPV4 inhibition. We also show for the first time, the successful gene editing of human herniated and degenerated AF cells (Pfirrmann grade 2 to 4) via CRISPR-Cas9 for the purpose of knocking out the TRPV4 gene and investigating its role. While the CRISPR-based technology allowed us to confirm the role of TRPV4 in regulating IL6 and IL8, it could be used in future studies to modulate other molecular targets involved in IVD pathologies [33].

Our model exhibits features of stretch-induced low-grade mechanoflammation, representative of early-stage AF injury [12]. In agreement with previous studies [13,14,15,16,17], the hyperphysiological cyclic stretching of AF cells induced an increase in gene expression of IL6, IL8, COX2 and MMP1. This pro-inflammatory response was transient and decreased with increasing stretching durations. Interestingly, Pratsinis and colleagues have similarly reported a bell-shaped expression of pro-inflammatory genes with increasing stretching durations in AF cells [17]. This is compatible with a single continuous loading event, rather than a repetitive loading regime. The reduction of COL2A1 and ACAN mRNA in response to cyclic stretching, also previously reported by another study [13], confirms the degenerative purpose of our hyperphysiological stretching regime. The moderate but significant increase in PGE2 release caused by hyperphysiological cyclic stretching further confirms the COX2 gene expression increase. The lack of augmentation of IL6 and IL8 release in response to stretching could be due to the existence of other rate limiting steps in the involved pathways, or unknown feedback mechanisms that prevented the translation of the stretch-upregulated IL6 and IL8 mRNAs. In fact, RNA-binding proteins can bind to the adenylate-uridylate (AU)-rich elements in the 3′-untranslated regions of the inflammatory mediators’ mRNAs in order to destabilize them, or induce translational silencing to resolve acute inflammation [34,35]. Our finding that hyperphysiological cyclic stretching significantly increased the phosphorylation of p38 is in agreement with the study of Pratsinis et al. [17]. However, in contrast to this report, the activation of ERK and JNK was not statistically significant. With the increased gene expression of IL6, IL8, COX2 and MMP1, the reduced gene expression of COL2A1 and ACAN, the increased PGE2 release and the activation of p38, we established a model of acute pro-inflammatory response to hyperphysiological stretching.

The novel finding that TRPV4 inhibition significantly reduces the stretch-induced gene expression of IL6, IL8 and COX2, but not MMP1, proposes a role of TRPV4 in mediating, at least in part, mechanoflammation in AF cells. It is to be noted that the link between TRPV4 and these pro-inflammatory mediators finds an echo in studies with other organs and tissues. The stretch-induced release of IL6 by mouse lung epithelia cells was modulated by TRPV4 inhibition [36]. In human epidermal keratinocytes, TRPV4 blocking reduced IL6 and IL8 production caused by gamma irradiation [37]. Additionally, the stretch-induced upregulation of COX2 expression was decreased by treatment with a TRPV4 antagonist in human periodontal ligament cells [38]. The reduction of stretch-induced PGE2 release in the presence of the TRPV4 antagonist further confirms the reduction in COX2 gene expression. Moreover, these results are in agreement with a study reporting that the increase in PGE2 release, upon hypo-osmotic stress in porcine chondrocytes, is TRPV4 dependent [39]. Interestingly, stretch-induced p38 phosphorylation was also significantly attenuated by the treatment with the TRPV4 inhibitor. While this finding is new in AF cells, it is corroborated by other studies highlighting the role of p38 in TRPV4-mediated (mechanically induced) inflammation or pain in other organs [36,37,40]. Since we did not analyze the expression of inflammatory mediators in non-stretched cells treated with GSK2193874, it is not possible to know whether their decrease already occurs without stretching. Despite using different concentrations of the TRPV4 antagonist in inhibition experiments, it was not clear whether the observed responses were dose dependent. We attribute this to the moderate effects and variability between different human donors.

In a second part of the study, we conducted experiments with CRISPR-Cas9 TRPV4 KO cells. Although two other research groups have previously reported the gene editing of human nucleus pulposus cells via CRISPR [41,42], we are the first, to our knowledge, to successfully implement CRISPR-Cas9 gene editing in human-degenerated AF cells. TRPV4 KO completely reversed the stretch-induced upregulation of the IL8 gene expression, thus confirming the role of TRPV4 in the mechanoregulation of IL8. TRPV4 KO further tended to reduce the stretch-induced increase in IL6 mRNA, in agreement with the TRPV4 inhibition data. Surprisingly, hyperphysiological cyclic stretching did not upregulate COX2 mRNA and PGE2 release in transduced NT cells. In addition, the basal concentrations of pro-inflammatory mediators were higher in the transduced cells compared to the naive cells, thus suggesting that the used transduction protocol might provoke cellular stress. The fact that TRPV4 KO stretched cells released significantly less IL6 compared to the NT stretched cells strengthens the hypothesis that TRPV4 regulates IL6 during mechanical loading. Interestingly, TRPV4 KO non-stretched cells also released less IL8 compared to NT non-stretched cells, highlighting again a link between TRPV4 and IL8.

Although in vitro models are well suited to explore molecular and cellular mechanisms, our findings need to be confirmed at the IVD organ scale and in vivo. Many preclinical studies using TRPV4 inhibitors as a treatment for diverse diseases have already been conducted [40,43,44,45]. Recently, the TRPV4 antagonist GSK2798745 was administered to healthy volunteers and heart failure patients without any safety concern [46]. Preclinical and clinical studies investigating TRPV4 inhibition in the context of DDD and LBP are currently lacking. If the mechanoinflammatory role of TRPV4 is further confirmed in vivo, TRPV4 may become a therapeutic target for novel treatments of LBP. This novel type of “mechanomedicine” may benefit patients with IVD pathologies caused by aberrant mechanical loading and hyperphysiological stretching. Mechanical stressors such as impact, heavy lifting, muscle activations, and work/lifestyle factors (e.g., vibration exposure, gait, and posture) [47] are associated with IVD degeneration or injury [2]. In particular, AF disruption is commonly linked to LBP and disability [12]. The healing process following AF injury includes inflammation, the recruitment of immune cells, cell proliferation, the formation of granulation tissue and matrix remodeling [12]. However, if initial inflammation is not resolved, it can become chronic and cause degeneration and pain [12]. The pro-inflammatory mediators investigated in this study downstream of TRPV4 have previously shown to be clinically relevant. Human subjects with LBP were found to display significantly higher levels of IL6 in serum compared to control subjects [48]. Moreover, higher serum levels of IL6 and IL8 mRNA and protein were found in patients with more severe LBP [49,50]. Cytokines can further induce COX2 expression and the subsequent synthesis of PGE2 [51]. Higher levels of COX2 and PGE2 have been found in herniated discs compared to controls [52,53]. Finally, MAPKs are important stress and inflammation regulators in the IVD, and p38 was shown to control the expression of IL6, IL8 and COX2, among other mediators [54]. Currently, p38 inhibitors are being investigated to treat inflammation-related diseases [54]. The mechanisms of action of the current anti-inflammatory treatments of LBP remain unclear [10]. By targeting a specific mechanosensitive marker such as TRPV4, it might be possible to specifically address mechanoflammation and increase effect sizes. Due to its polymodal nature, a targeted and localized silencing of TRPV4 in the AF, e.g., via gene editing, might be desirable in the future. The lentiviruses used in this study randomly integrate in the genome, but they could be replaced in clinical applications by adeno-associated viruses, which integrate into safe harbor regions [55].

## 5. Conclusions

“Mechanoflammation” caused by hyperphysiological cyclic stretching was mitigated by TRPV4 inhibition with the specific GSK2193874 antagonist, thus revealing the novel mechanoinflammatory role of TRPV4 in human primary AF cells. In fact, the stretch-induced upregulation of IL6, IL8 and COX2 gene expression, PGE2 release and p38 phosphorylation were significantly reduced by TRPV4 inhibition. We further report the use of CRISPR-Cas9 technology to successfully knock out TRPV4 in human AF cells. Remarkably, increases in IL8 mRNA levels caused by hyperphysiological cyclic stretching were completely prevented by TRPV4 KO. TRPV4 KO further reduced the release of IL6 and IL8. Our results thus suggest that TRPV4 mediates stretch-induced inflammation possibly via the activation of the p38 MAPK pathway. Future pharmacological or gene-editing therapies to treat DDD and LBP might thus target TRPV4 or the molecular mediators interacting with it in the signaling pathway.

## Figures and Tables

**Table 1:** Patient characteristics: F = female; M = male; DDD = degenerative disc disease; L = lumbar; C = cervical.

N°	Age	Sex	Diagnosis	Disc Level	Pfirrmann Grade
1	74	M	Herniation	L4/5	3
2	71	M	Herniation	L4/5	3
3	76	F	DDD	L5/S1	4
4	56	F	DDD	C6/7	3
5	40	F	Herniation	C5/6	2
6	52	F	Herniation	L4/5	3
7	15	M	DDD	L4/5	4
8	75	F	DDD	L3/4	3
9	46	M	Herniation	L4/5	4

## Supplementary Materials

The following are available online at https://www.mdpi.com/2073-4409/9/7/1736/s1, Figure S1. Immunocytochemistry of NT and KO cells from two additional donors; and NT cells without primary antibody.
