# Supplementary material for: TRPV4 Inhibition and CRISPR-Cas9 Knockout Reduce Inflammation Induced by Hyperphysiological Stretching in Human Annulus Fibrosus Cells
Source: Cells. 2020 Jul 21;9(7):1736. doi: 10.3390/cells9071736 (PMC7407144; doi:10.3390/cells9071736)
Supplement: Supplementary file 1 [file cells-09-01736-s001.pdf]

Supplementary Information

TRPV4 inhibition and CRISPR-Cas9 knockout reduce inflammation induced by hyperphysiological stretching in human annulus fibrosus cells

Elena Cambria, Matthias J. E. Arlt, Sandra Wandel, Olga Krupkova, Wolfgang Hitzl, Fabian S. Passini, Oliver N. Hausmann, Jess G. Snedeker, Stephen J. Ferguson, Karin Wuertz-Kozak

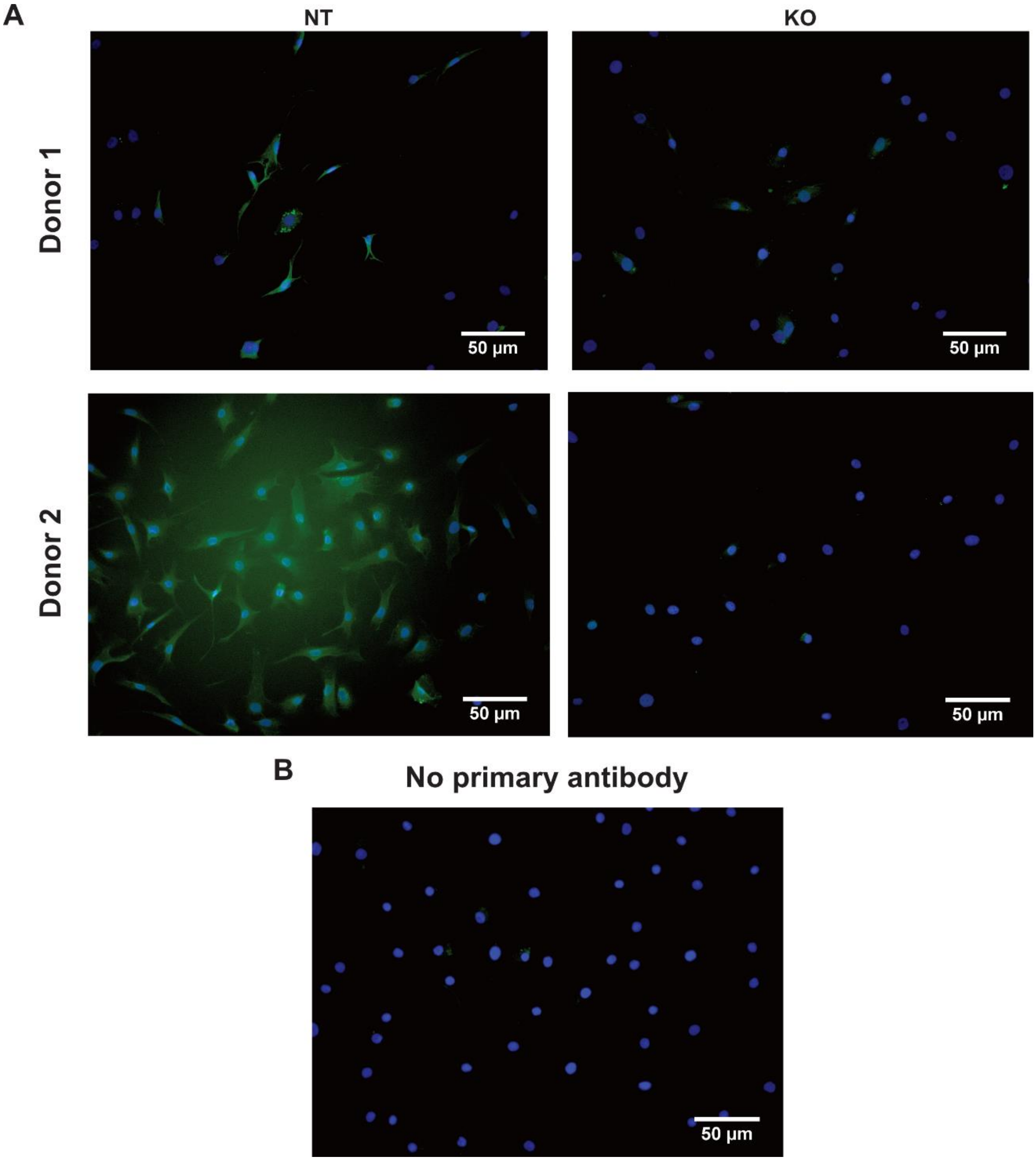

Figure S1. (**A**) Immunocytochemistry of (**A**) NT and KO cells from two additional donors; and (**B**) NT cells without primary antibody; green = TRPV4, blue = DAPI; scale bars = 50 µm.
